# Hydrogen Bonds Are Never of an “Anti-electrostatic” Nature: A Brief Tour of a Misleading Nomenclature

**DOI:** 10.1021/acs.jpclett.4c00779

**Published:** 2024-04-18

**Authors:** Carlos Martín-Fernández, M. Merced Montero-Campillo, Ibon Alkorta

**Affiliations:** † School of Chemistry, University of St Andrews, Purdie Building, St Andrews KY16 9ST, U.K.; ‡ Departamento de Química (Módulo 13, Facultad de Ciencias), Campus de Excelencia UAM-CSIC, Universidad Autónoma de Madrid, 28049 Madrid, Spain; § Instituto de Química Médica (CSIC), 28006 Madrid, Spain

## Abstract

A large amount of scientific works have contributed through the years to rigorously reflect the different forces leading to the formation of hydrogen bonds, the electrostatic and polarization ones being the most important among them. However, we have witnessed lately with the emergence of a new terminology, anti-electrostatic hydrogen bonds (AEHBs), that seems to contradict this reality. This nomenclature is used in the literature to describe hydrogen bonds between equally charged systems to justify the existence of these species, despite numerous proofs showing that AEHBs are, as any other hydrogen bond between neutral species, mostly due to electrostatic forces. In this Viewpoint, we summarize the state of the art regarding this issue, try to explain why this terminology is very misleading, and strongly recommend avoiding its use based on the hydrogen bond physical grounds.

Noncovalent interactions (NCIs)^1^ of different kinds are many times at the very heart of conformational preferences^2,3^ and behind the existence of ubiquitous intermolecular assemblies for different functionalities or purposes.^4−8^ The importance of hydrogen bonds (HBs) in the Watson–Crick–Franklin motifs,^9^ the HBs and stacking interactions in biomaterials design,^10,11^ or the NCIs in artificial structures such as MOFs and COFs^12^ or graphene engineering^13^ is well known, to cite only some representative examples beyond the awesome properties of water. In this sense, despite the recognized difficulty in accurately describing them because of the energy range of these interactions and their complex physical background,^14^ noncovalent interactions (with different names) have been a key topic in chemistry from the 19th century^15^ up to the very diverse and rich present,^16^ and the HB in particular has been a part of the common chemical vocabulary since its inclusion in one of the most famous chemistry textbooks of all time.^17,18^

The boundary between covalent and noncovalent bonds has never been clear,^19^ in particular in tricky cases such as intramolecular interactions or strong dative bonds. If we want to simplify it to its minimum expression and leave aside ionic interactions, covalency and noncovalency are different regimes within chemical bonding, where covalency is devoted to a large degree of electron sharing between the involved species, and noncovalency is usually reserved to weakly interacting species, most commonly closed-shell systems, in which the interacting subunits essentially keep their structural identities. However, there is no clear-cut line even under the NCI label regarding the limit of the strength of the interaction, as exemplified by the particular case of short hydrogen bonds like the [F–H···F]^−^ one^20^ and the extensive research devoted to analyzing the changing nature of HBs from traditional to proton shared to ion pair.^21^ To add even more complexity, the effect of weak NCIs in the properties of a given system may be equal or even larger than those of strong NCIs.^22^ Nonetheless, both weak and strong noncovalent interactions share some key features: they trigger changes in the properties of the interacting subunits, induce a certain amount of geometrical distortion, and lead to cooperativity or anticooperativity effects, all of them to different extents depending on the type of NCI.^23^ The IUPAC has added to their guidelines and scientific glossaries the main physicochemical descriptors of NCIs less present in general textbooks, such as halogen bonds^24^ or chalcogen bonds;^25^ still, the *old* hydrogen bonds are the most famous ones, and there are many reasons behind this stardom within the noncovalency world.

Based on Werner’s theory on ammonium chlorides and hydroxides,^26^ the existence of hydrogen bonds was suggested already in 1912 by Winmill and Moore,^27^ who illustrated the system formed by trimethylammonium and water as a weak electrolyte where hydrogen is still attached to the OH group while interacting with nitrogen. According to Arunan and collaborators,^28^ who in turn quote Pauling, Latimer and Rodebush in 1920 might have been among the first ones to use the term “hydrogen bond”.^29^ Lewis in 1923 also claimed that Huggins suggested a similar description of the HB already in 1919.^30,31^ The Arunan report for the IUPAC, to which one of us has contributed, summarizes the physical forces governing hydrogen bonds with this statement: “It is well accepted that hydrogen bonding has contributions from electrostatic interactions between permanent multipoles, polarization, or induction interactions between permanent and induced multipoles, dispersion arising from instantaneous multipoles-induced multipoles, charge-transfer-induced covalency, and exchange correlation effects from short-range repulsion due to overlap of the electronic distribution.” The complex hydrogen bond nature requests this long list for an accurate description, but the large contribution of the electrostatic part is out of the question when discussing one of the main features of this interaction: directionality,^32^ which rules out dispersion as the leading voice (although relevant in very weak cases). Electrostatics and directionality are therefore the essence of the hydrogen bond, and this is particularly true for donors, such as oxygen, fluorine, and nitrogen, a set of very electronegative atoms that give rise to strong hydrogen bonds.

Following the publication of the IUPAC report on the definition of hydrogen bonds, several critical voices have emerged through the years, recently pointing out that besides the classical protonic form, other forms of hydrogen bonding must be included in the definition^33^ or, importantly, that covalency is dominant in HBs and electrostatic effects are “found to play only a minor role”.^34^ In this context of questioning the entire universe of hydrogen bonds, which is completely legitimate, an eye-catching name, “anti-electrostatic hydrogen bonds”, accompanied by a quite settled acronym, AEHBs, made its appearance.^35^ The AEHB nomenclature was applied to HBs formed between anionic species, a name that seems to deny the main role of electrostatics in the formation of these hydrogen bonds. Our interpretation is that the nomenclature arose because it was found to be shocking that equally charged systems bound by HBs could exist, despite the large Coulombic repulsion between the species. Thus, it would seem that these hydrogen bonds defy electrostatic repulsion and still somehow form. However, we must note that, in the covalent world, the existence of such bonded multiply charged species is not in doubt. In fact, there is a plethora of examples of this latter kind, starting by very small systems like N_2_^2+^,^36^ with a significant lifetime, or the paradigmatic He_2_^2+^,^37^ whose stability was predicted by Pauling more than 50 years before its discovery,^38^ in contrast with the very weak van der Waals governed neutral helium dimer.^39^ The existence of diatomic dications is beautifully rationalized by Schröder and Schwarz through a comparison of potential energy curves depending on the ionization energies of species A and B interacting to form an AB^2+^ molecule,^40^ thus avoiding the Coulombic explosion scenario. The barrier preventing dissociation is strongly dependent on the nature of the system, that of the dicationic helium dimer being around 145 kJ/mol, although fragmentation is exothermic by more than 900 kJ/mol.^37^ In general terms, we can state without a doubt that the field of covalently bonded multiply charged ions has been and continues to be the subject of intense research.^41−44^

The same principles that apply for covalent bonds joining charged species are valid for noncovalently charged species as well. These species could exist if the NCIs involved are strong enough, although in general they are more challenging to form in the gas phase with respect to covalent cases. There is a variety of cases reported in the gas phase linked by hydrogen bonds not only for small systems but also for larger ones, such as singly charged peptides formed via electrospray ionization,^45^ betaine clusters,^46^ or bisulfate anions and organophosphates anions confined in cyanostar macrorings.^47,48^ Other kinds of NCIs can stabilize charged dimers in the gas phase as pointed out by several theoretical studies, like spodium bonds,^49^ aerogen bonds,^50^ chalcogen bonds,^51^ or the more explored halogen bonds.^52^ Some of these theoretical studies are motivated by the existence of similar interactions in condensed phases, for instance, crystals reported in the CSD or in solution,^52−55^ finding a connection between stable systems in the solid state and metastable species in the gas phase with the corresponding characterization of the barrier preventing dissociation (see Figure 1), as in the paradigmatic He_2_^2+^ case. Charged species bound by NCIs in the solid state are less surprising, as counterions or the solvent are there to compensate electronic repulsion,^56−58^ a key aspect we will come back to shortly.

**Figure 1:**
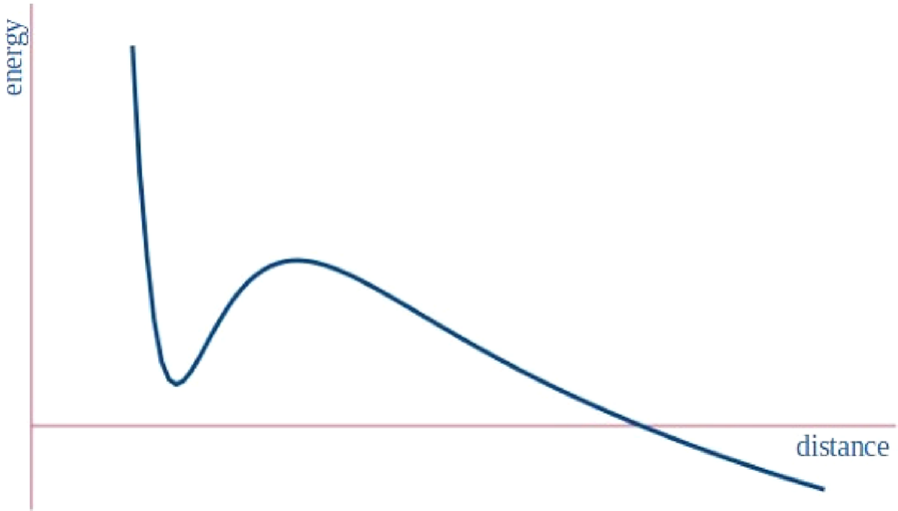
Energy vs distance profile of a generic metastable charged dimer linked by a noncovalent interaction. It presents a local minimum with positive interaction energy with a non-negligible barrier preventing dissociation. The inclusion of counterions or solvent conditions may lead to a stable minimum.

Together with studies noticing the peculiarity of anion–anion and cation–cation systems apparently defying Coulombic forces thanks to hydrogen bonds, the corresponding nomenclature for these HBs started to emerge (Table 1). In this sense, Novoa et al. use the term “inter-anion” for hydrogen bonds in their work dealing with chains of HC_2_O_4_^–^ ions,^59^ and just one year later Steiner in 1999 stated that inter-anion HBs are fundamentally equal to classic HBs.^60^ It is striking to find that more than 20 years later the “inter-anion” or “inter-cation” hydrogen bonds seem to have been replaced by the AEHB terminology mentioned before. The noncovalent interactions field is not the only one to appeal to “anti-electrostatics” as research on ionic liquids also uses this term,^61^ although it is gradually being replaced by “anti-static”,^62^ since it is applied to the quality of ionic liquids to work as anti-static agents in polymers.

**Table 1:** A Set of Terms Related to Hydrogen Bonds between Charged Systems That Have Received Terminological Distinctions in the Literature

Kind of hydrogen bond	Year^a^	Reference
Ionic hydrogen bond	1983	(63)
Inter-anionic hydrogen bond	1992	(64)
Inter-cationic hydrogen bond	1994	(65)
Inter-ionic hydrogen bond	2004	(66)
Cation–anion hydrogen bonds	2011	(67)
Antielectrostatic hydrogen bond	2014	(68)

a Search considering the first appearance of the term in the title, keywords or abstract in indexed articles, according to Reaxys database.^69^ Note that “year” does not necessarily indicate the first time the term was used, but only the earliest entry according to the previously mentioned searching constraints.

Regardless of the rigorous work carried out by many of the researchers coining the AEHBs acronym, we find it very misleading to say, to cite one example, that a tetraanionic [HPO_4_···HPO_4_]^4–^ dimer is “highly anti-electrostatic”.^35^ It is clear that there will be a strong Coulombic repulsion in a complex with a total charge of 4–, as it is clear that the dimer exists in the solid state (CSD refcode FUJPEF) thanks to the stabilizing presence of the counterions, but this does not mean that the HBs that hold it together are anti-electrostatic (in the interpretation of the term described above). To complicate things, the interactions in the crystal will be a balance of attractive and repulsive electrostatic forces as well as others. To really find out if electrostatics is against the formation of hydrogen bonds (which is what the AEHBs name evokes), it is crucial to study the role of the hydrogen bonds without any other environmental effects entering the game or, if we prefer to say it differently, to quantify the intrinsic stability of the system, avoiding other factors affecting global stability. In this sense, studies in condensed phases are less likely to quantifying the contribution of the solvent or the crystalline phase. Moreover, hydrogen bonds are not (obviously) the only reason responsible for binding, even in systems with a net charge equal to zero. For instance, it has been also shown through the study of the Hansen solubility parameter that non-water-soluble DNA segments bind each other because dipolar interactions are just as important as the contributions arising from exchange, to the point that “the emphasis on hydrogen bonding *alone* as being the decisive factor in DNA base binding should be revised.”^70^

Experimental^60,71,72^ and theoretical^73−76^ evidence strongly supports the fact that inter-anionic or inter-cationic HBs are conventional HBs. Among the studied systems, the polemic case of interacting hydrogenoxalate anions in the solid state has been analyzed in detail.^59^ On the experimental side, the analysis of the O–H and the H···O distances are fully in line with values for similar strong hydrogen bonds.^60^ For the same system in solution, the variation of the chemical shift of the acidic proton with increasing concentration responds to the expected behavior of a classical H-bonding isotherm, which can be adjusted through a least-squares fitting procedure to a polynomic expression that takes into account the different *n*-mer species in solution.^71^ The electron density extracted from accurate low-temperature X-ray diffraction data shows that the molecule in the crystal undergoes a very strong polarization, making the vacuum model not enough to predict the binding observed in the crystal.^74^ On the other hand, the analysis of the electric field in charged complexes stabilized by HBs demonstrates that the electrostatics associated with the HBs is the essential reason for the formation of these complexes, as exemplified by the very representative phosphate–phosphate complex case.^77^ If the H···O distance increases slightly with respect to the equilibrium position, the electron density locked in the bonding region is enough to induce attractive forces and restore the equilibrium; however, at longer distances, the lock triggered by the HB is surpassed by the repulsive electrostatic forces (which are never out of the picture), and dissociation takes place. Importantly, the electrostatic interaction energy of the hydrogen bonds obtained from the integration of the electrostatic moments on the basins of the interacting O and H atoms exhibits a clear correlation with the interaction energy.^77^ If energy decomposition techniques are used to analyze the contributions of the different energy terms that make up the interaction, and often regardless of the specific technique used, electrostatics is still the main contribution not only in HBs but also in other NCIs.^78−80^ This effect is particularly noticeable if we compare the electrostatic contribution in HB-bonded charged systems at the minimum with respect to that of the transition state of the dissociation process. The electrostatic contribution is repulsive in both cases, as expected for an interaction between equally charged species, but it is less so in the minimum than in the TS, a somewhat unexpected result since the species are closer together in the minimum, and the repulsion should be larger. The reason behind this difference, simply put, is that in the minimum there is an attractive electrostatic component from the HB that is not present in the TS.^73,78^ In a very recent work, Chen and collaborators studied with the AMOEBA polarizable force field halogen and hydrogen bonds between anion–anion complexes,^80^ finding that the nonmonotonic characteristic of the electrostatic interaction can be well interpreted by classical electrostatic theory and interanionic and conventional halogen and hydrogen bonds behave equally. An example that illustrates this idea is the electric field maps of two related systems such as the neutral pyridine boronic acid dimer and the charged pyridine boronic acid dimer, which are almost identical in the interbonding region, and there is a need to explore regions far from the HBs to find the main differences.^53^ We provide here the electric field of the neutral and protonated carbamic acid dimers, as well as that of the neutral and deprotonated carbonic acid dimers (Figure 2), as additional examples. The lines represented in the map allow the visualization of the electrostatic forces acting on a given molecular region. The electrostatic attraction region, where lines start in H atoms and electron density belongs to O atoms, exist in neutral and charged dimers. Comparing neutral and charged dimers, the electric field in the intermolecular region is very similar, particularly in the anion dimer where the geometry is much alike to that of the neutral partner. Again, the lines behave notably different around the substituents of the carboxylic groups, evidencing the effect of ionization on the dimers.

**Figure 2:**
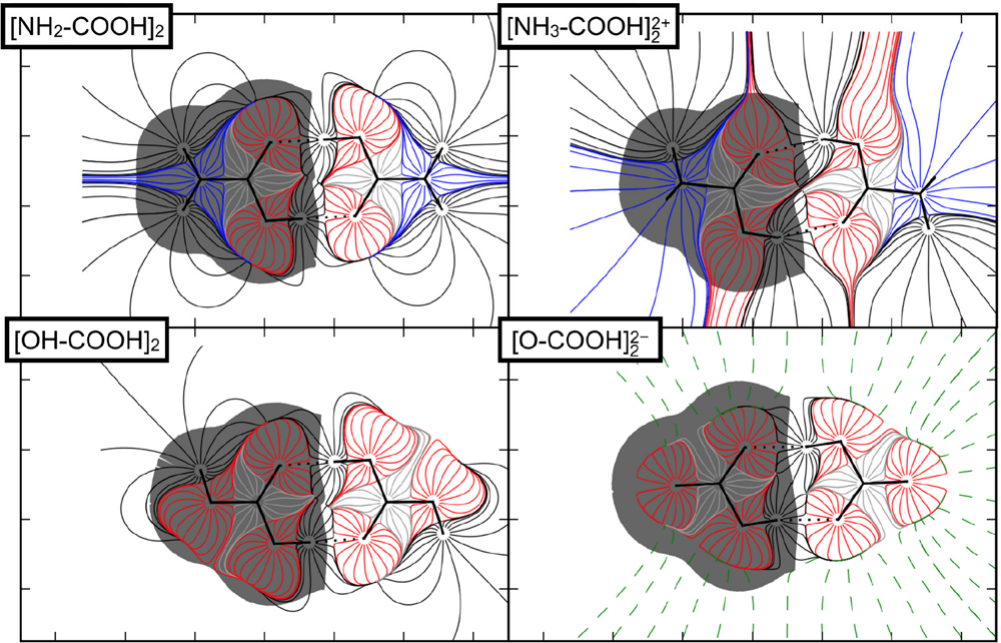
Electric field maps: first row, carbamic acid (NH_2_COOH)_2_ dimer and protonated carbamic acid dimer; second row, carbonic acid (OHCOOH)_2_ dimer and deprotonated carbonic acid dimer. Basin color code: N, blue; O, red; C, gray; H, no color. The volume of one of the monomers forming the dimer is indicated as a shaded region, with this volume being defined within the QTAIM framework.^81^ Structures were optimized at the M06-2X/aug-cc-pVTZ level. The maps were calculated from the electrostatic potential, obtained with AIMAll^82^ and represented with the Matplotlib library.^83^

Finally, we remark once more on the key effect of the environment when discussing these equally charged noncovalent dimers. It has been shown in a significant number of works that metastable charged complexes in the gas phase with positive binding energies are stabilized in polar solvents or by adding counterions.^84−91^ Clearly, the “anti-electrostatic” terminology is even less appropriate when considering these systems in the condensed phase.

As a summary of all the ideas discussed in this Viewpoint, we would like to highlight the following key points: (i) Covalently bonded charged species are never considered “anti-electrostatic”; nonetheless, their potential energy curves with respect to dissociation are very similar to those found for hydrogen bonded charged complexes. (ii) Hydrogen bonds are, from all points of view, essentially of the same nature in neutral and charged complexes. (iii) Electrostatics is a driving force in the formation of hydrogen bonds, and consequently, it is fully present in noncovalently bound multiply charged systems. (iv) The adjective “anti-electrostatic” is especially misleading in the case of HBs, as electrostatics is never out of the interplay between forces, where the medium plays a stabilizing role. (v) The nomenclature “interanion/intercation” (or interanionic/intercationic) hydrogen bonds is well established in the literature and avoids possible confusion regarding the true nature of hydrogen bonds. Although this discussion may seem only a semantic question, it is indisputable that scientific concepts can be misinterpreted if the vocabulary does not rigorously adjust to the essence of what they are intended to describe, an effort that has been carried out since time immemorial by the various international scientific bodies that ensure the correct definition of these concepts. These conclusions could apply as well to other noncovalent interactions^92,93^ if they share physical grounds with the hydrogen bond.
